# Antidiabetic treatment with gliptins: focus on cardiovascular effects and outcomes

**DOI:** 10.1186/s12933-015-0294-0

**Published:** 2015-09-29

**Authors:** Enrique Z. Fisman, Alexander Tenenbaum

**Affiliations:** Sackler Faculty of Medicine, Tel Aviv University, Ramat Aviv, 69978 Tel Aviv, Israel; Cardiovascular Diabetology Research Foundation, 58484 Holon, Israel; Cardiac Rehabilitation Institute, Sheba Medical Center, 52621 Tel Hashomer, Israel

**Keywords:** Antidiabetic treatment, Cardiovascular outcomes, Dipeptidyl peptidase-4 (DPP4) inhibitors, Gliptins, Heart failure, Incretins, Ischemic heart disease, Major adverse cardiovascular events (MACE), Type 2 diabetes mellitus

## Abstract

The traditional oral pharmacological therapy for type 2 diabetes mellitus (T2DM) has been based on the prescription of metformin, a biguanide, as first line antihyperglycemic agent world over. It has been demonstrated that after 3 years of treatment, approximately 50 % of diabetic patients could achieve acceptable glucose levels with monotherapy; but by 9 years this had declined to only 25 %. Therefore, the implementation of a combined pharmacological therapy acting via different pathways becomes necessary, and its combination with a compound of the sulfonylurea group was along decades the most frequently employed prescription in routine clinical practice. Meglitinides, glitazones and alpha-glucosidase inhibitors were subsequently developed, but the five mentioned groups of oral antihyperglycemic agents are associated with variable degrees of undesirable or even severe cardiovascular events. The gliptins—also called dipeptidyl peptidase 4 (DPP4) inhibitors—are an additional group of antidiabetic compounds with increasing clinical use. We review the status of the gliptins with emphasis on their capabilities to positively or negatively affect the cardiovascular system, and their potential involvement in major adverse cardiovascular events (MACE). Alogliptin, anagliptin, linagliptin, saxagliptin, sitagliptin, teneligliptin and vildagliptin are the compounds currently in clinical use. Regardless differences in chemical structure and metabolic pathways, gliptins as a group exert favorable changes in experimental models. These changes, as an almost general rule, include improved endothelial function, reduction of inflammatory markers, oxidative stress ischemia/reperfusion injury and atherogenesis. In addition, increased adiponectin levels and modest decreases in lipidemia and blood pressure were reported. In clinical settings, several trials—notably the longer one, employing sitagliptin, with a mean follow-up period of 3 years—did not show an increased risk for ischemic events. Anyway, it should be emphasized that the encouraging results from basic science were not yet translated into clinical evidence, probably due the multiple and pleiotropic enzymatic effects of DPP4 inhibition. Moreover, when employing saxagliptin, while the drug was not associated with an augmented risk for ischemic events, it should be pinpointed that the rate of hospitalization for heart failure was significantly increased. Gliptins as a group constitute a widely accepted therapy for the management of T2DM, usually as a second-line medication. Nonetheless, for the time being, a definite relationship between gliptins treatment and improved cardiovascular outcomes remains uncertain and needs yet to be proven.

## Review

### Background

The traditional oral pharmacological therapy for type 2 diabetes mellitus (T2DM) has been based on the prescription of metformin as first line antihyperglycemic agent world over. This biguanide derivate is the most widely prescribed drug to manage glucose metabolism in individuals with T2DM and is still recommended, in conjunction with lifestyle modification, as a first line medication in the joint guidelines of the American Diabetes Association and the European Association of the Study of Diabetes [1]. It has been demonstrated that after 3 years of treatment, approximately 50 % of diabetic patients could achieve acceptable glucose levels with monotherapy; but by 9 years this had declined to only 25 % [2]. Therefore, the implementation of a combined pharmacological therapy acting via different biochemical pathways becomes necessary, and its combination with a compound of the sulfonylurea group was along decades the most frequently employed prescription in routine clinical practice [3]. While it is generally considered a relatively safe drug, increased mortality associated with the use of metformin was reported during long [4] and even short-term follow-up [5]. Appearance of lactic acidosis has been reported as well, especially in the elderly and in patients with liver failure [6, 7].

Cardiovascular derangement has been widely described for most sulfonylureas, which exert their action by closing the ATP-dependent potassium channels; this feature is accountable for both the insulinotropic and the undesirable effects on the heart. During myocardial ischemia, sulfonylureas partially inhibit channels opening, avoiding so the required hyperpolarization that protects the cell by impeding calcium influx (the so-called “ischemic preconditioning”) [8, 9]. In a population of coronary patients, data from our laboratory indicated a huge increased all-cause crude mortality over a mean 7.7-year follow-up in diabetics on combined treatment with metformin and glibenclamide; figures on mortality in this group almost quadrupled those of nondiabetic coronary patients [10]. Similar findings have been reported in a general diabetic population [11], and the increased morbidity and mortality of this combined therapy was recently confirmed in a large nationwide Danish study [12]. Glinide compounds like repaglinide—which also act by closing the ATP-dependent potassium channels—appear to be associated with a similar risk of adverse cardiovascular sequelae than sulfonylureas [13].

Regarding glitazones, these compounds have been associated with a higher risk of developing stroke, heart failure and all-cause mortality [14–17]. These excess risks were largest in people aged 65 years or older, and especially in patients treated with rosiglitazone [15].

Concerning alpha-glucosidase inhibitors, voglibose was found to improve endothelial function in patients with type 2 diabetes [18] and miglitol enhanced postprandial endothelial function in patients with acute coronary syndrome and new-onset postprandial hyperglycemia [19]. Moreover, acarbose has been postulated as a potential agent for coronary disease secondary prevention [20, 21]. Despite these promising perspectives, no unequivocal benefits have been established since in a recent large intention-to-treat analyses, acarbose was associated with a higher risk of development of any cardiovascular event, heart failure, and ischemic stroke compared to metformin [22].

As stated above, the five mentioned groups of oral antihyperglycemic agents—biguanides, sulfonylureas, meglitinides, glitazones and alpha-glucosidase inhibitors—are associated with variable degrees of undesirable or even severe cardiovascular events. Moreover, the adequate treatment in case of comorbidities is not defined [23]. Taking these facts into consideration, the Food and Drug Administration (FDA) recommended that in order to establish the safety of a new antidiabetic therapy for T2DM, the manufacturers or sponsors should demonstrate that the new drug will not result in an unacceptable increase in cardiovascular risk and the trials population should also include high risk patients. Other criteria included trials duration of at least 2 years [24]. A sixth pharmacological group, the gliflozins, constitutes a new category, and its yet unknown long-term cardiovascular outcome is currently being investigated [25]. The gliptins—also called dipeptidyl peptidase-4 (DPP4) inhibitors—are an additional group of antidiabetic compounds with increasing worldwide clinical use since the first of them, sitagliptin, got the FDA approval in 2006 [26]. In this context, the purpose of the present article is to review the status of the gliptins currently in clinical use, with special emphasis on their capabilities to positively or negatively affect the cardiovascular system, and their potential involvement in major adverse cardiovascular events (MACE).

### Biochemistry

Dipeptidyl peptidases are a family of several complex proteases with similar chemical structure; the biological roles and identity of their endogenous substrates remains poorly understood for the majority of them [27]. Therefore, a cautious evaluation of the selectivity and specificity of any pharmacological compound used to inhibit DPP4 activity is required [28]. For the time being, DPP4 is the best known member of the family and acts as a membrane-anchored cell surface peptidase transmitting intracellular signals through a short intracellular tail. In humans, the DPP4 gene is located on chromosome 2 locus 2q24.3 and composed of 26 exons that encode a protein of 766 amino acids [29]. DPP4 is widely expressed in several cell types, particularly in exocrine glands and absorptive epithelia. It is mainly found in the brush borders of epithelial cells of the proximal convoluted tubules in the kidney, in the small and large intestine, prostate tissue, hepatocytes, fibroblasts and in activated leukocytes [30–33], preferentially cleaving peptide hormones containing a position two alanine or proline [27].

The incretins are gut-derived hormones, constituents of the glucagon superfamily, released in response to nutrient ingestion, mainly sugars and fat. They put forth a wide range of effects, including stimulation of pancreatic insulin secretion in a glucose-dependent manner and play a central role in local gastrointestinal and whole-body physiology. The principal incretins are the glucose-dependent insulinotropic polypeptide (GIP) and the glucagon-like peptide-1 (GLP-1), representing the endogenous physiological substrates for DPP4 activity [34, 35]. GIP is secreted from the L-cells of the distal ileum and colon and GLP-1 from the K-cells in the duodenum and jejunum [36]. GLP-1 is stronger than GIP regarding insulinotropic activity, and their biological activity is cumulative. GIP has a half life of approximately 7 min, much longer than the 2 min documented for GLP-1 [27, 37, 38]. Therefore, taking into consideration the DPP4 mechanism of action, its pharmacological inhibition by gliptines will avoid interaction with its substrates, increasing plasmatic GIP and GLP-1 concentrations and stimulating insulin biosynthesis [39].

Gliptins comprise a diverse group of compounds, which from a pharmacokinetic point of view can be broadly divided into peptidomimetics and non-peptidomimetics. Both are competitive reversible inhibitors of the DPP4 substrate acting extracellularly. The former are nitrile-containing inhibitors that act on the DPP4 substrate involving production of a reversible covalent enzyme–inhibitor complex, which gradually and slowly dissociates from the substrate leading to a lasting inhibitory activity, even after the drug has been inactivated. This phenomenon explains why these compounds act for longer than the relatively short half-lives of most of them would suggest. On the contrary, non-peptidomimetic drugs yield to a non-covalent extracellular cross-talk with residues in the catalytic site of the DPP4 substrate, resulting in a strong and immediate inhibition [40–43]. It has been suggested that these differences between compounds may also be reflected in their cardiovascular effects [44]. The differences in the metabolic pathways are also expressed in the therapeutic doses, which may range from 5 mg for saxagliptin to 100 mg for sitagliptin, and in the dosing frequency. The currently most used compounds include alogliptin [45–48], anagliptin [49–52], linagliptin [44, 53–56], saxagliptin [57–59], sitagliptin [60], teneligliptin [61] and vildagliptin [62]. Data regarding approving authority, chemistry, half life, dosage and catabolic pathway are summarized in Table 1. The research regarding an additional drug, dutogliptin, was discontinued [63], while several another compounds, notably gemigliptin [64] (already approved in South Korea) are currently at diverse stages of development and evaluation. Trelagliptin, a compound that unlike other approved agents of its class—which are usually administered once daily—can be administered once weekly, has been recently approved in Japan [65], and studies on another long-acting drug, omarigliptin, are in progress [66].

**Table 1:** Gliptins summarized data on approving authority, pharmacokinetics, chemistry, half life, dosage and catabolic pathway

Compound	Approval	Peptidomimetic	Chemistry	Maximal half life, hours	Usual dose, mg	Main catabolic pathway
Alogliptin	FDA, 2013	–	Pyrimidinedione	<21	25 qd	Renal
Anagliptin	Japan, 2012	+	Cyanopyrrolidine	<4.5^a^	100 bid	Renal
Linagliptin	FDA, 2011	–	Xanthine	<40	5 qd	Biliary
Saxagliptin	FDA, 2009	+	Cyanopyrrolidine	<4^b^	5 qd	Renal
Sitagliptin	FDA, 2006	–	Beta-amino acid	<24	100 qd	Renal
Teneligliptin	Japan, 2012	+	Prolylthiazolidine	<24	20 qd	Renal
Vildagliptin	European Union, 2007	+	Cyanopyrrolidine	<4.5	50 bid	Renal

^a^In addition, <10 h of partially active metabolite

^b^In addition, <7 h of partially active metabolite

### Pharmacological interactions

In daily clinical practice, gliptins are almost usually prescribed together with other antidiabetic, antihypertensive and antihyperlipidemic agents. Therefore, it is of utmost importance to establish whether potential undesirable interactions may be present. No adverse events were disclosed in the co-administration with metformin [67–69], glibenclamide [70], glitazones [71, 72], and simvastatin [73]. Moreover, in an experimental murine model, the combination with valsartan improved both pancreatic beta-cell function and insulin sensitivity, with a reduction of the inflammatory and cell stress milieu [74]. It should be mentioned that some gliptins, like linagliptin and saxagliptin may be metabolized via the CYP3A4 or CYP3A4/5 pathways, and this could result in a diminished clearance of other drugs using the same pathways. In addition, a reduction in the dose of sulfonylureas is usually recommended when a gliptin is added, since its pharmacodynamic interaction with the sulfonylurea may result in a higher risk of hypoglycemia. Anyway, this issue does not seem to be of clinical relevance [75]. As a general rule, there is no great propensity for the gliptins to be involved in any significant drug interaction with commonly prescribed antidiabetic medicines [42, 43].

### Cardiovascular effects of currently used gliptins

#### Sitagliptin

The effects of this medication—a beta-amino acid—on cardiovascular outcomes in patients with T2DM have been recently reported by Green et al. in the Trial to Evaluate Cardiovascular Outcomes after Treatment with Sitagliptin (TECOS) [60]. This is a randomized double-blind open-label study including nearly 15,000 patients in which co-administration of either sitagliptin or placebo was implemented on top of their previous antidiabetic treatment. The included patients had T2DM and proven cardiovascular disease and were at least 50 years old, with glycated hemoglobin levels of 6.5–8.0 % when treated with stable doses of one or two oral antihyperglycemic agents (metformin, pioglitazone, or a sulfonylurea) or insulin (with or without metformin). Sitagliptin dose was of 100 mg daily, or 50 mg daily in cases of kidney dysfunction. A composite of cardiovascular death, nonfatal myocardial infarction (MI), nonfatal stroke, or hospitalization for unstable angina was established as primary cardiovascular outcome. The secondary composite cardiovascular outcome was defined as the first confirmed event of cardiovascular death, nonfatal MI, or nonfatal stroke. In addition, other secondary outcomes comprised occurrence of the individual components of the primary composite cardiovascular outcome, fatal and nonfatal MI, fatal and nonfatal stroke, death from any cause, and hospitalization for heart failure. The study showed that after a mean follow-up period of 3 years, the addition of sitagliptin to the conventional pharmacological treatment did not have a significant effect on rates of major adverse cardiovascular events or hospitalization for heart failure. This study confirmed the findings of an earlier pooled analysis of 25 randomised clinical trials totalizing—similarly to the TECOS trial—almost 15,000 patients, which did not indicate that treatment with sitagliptin increases cardiovascular risk in patients with T2DM [76].

Data from other studies reported several beneficial effects. Indices of glycemic control, such as hemoglobin A1c, glycated albumin, and 1.5-anhydro-d-glucitol were significantly improved after a 3-month treatment with sitagliptin, and serum adiponectin level was significantly increased without changes of body weight [77]. Endothelial function was improved as well [18]. It was also suggested that previous chronic treatment with sitagliptin may have cardioprotective effects in diabetic patients presenting with acute coronary syndrome [78]. Moreover, in patients who were at high risk of heart failure after acute coronary syndrome, sitagliptin exposure was not associated with an increased risk of de novo heart failure [79] or other adverse cardiovascular events [80].

#### Saxagliptin

Saxagliptin, a cyanopyrrolidine, was the second gliptin that obtained the FDA approval. A comprehensive study including about 16,500 patients followed for a median of 2.1 years and describing the cardiovascular outcomes of this medication was reported by Scirica et al. in the SAVOR-TIMI 53 trial [58]. Patients with T2DM with documented or at risk for cardiovascular events were randomly assigned to receive saxagliptin or placebo. The usual dose was 5 mg daily, and the primary end point a composite of cardiovascular death, MI or ischemic stroke, and the main secondary end point a composite of cardiovascular death, MI, stroke, hospitalization for unstable angina, coronary revascularization, or heart failure. The study demonstrated that saxagliptin did not increase or decrease the rate of ischemic events. Subsequently, an analysis of pooled data from 20 clinical trials comprising over 9000 patients with T2DM confirmed that the drug was not associated with an increased risk for ischemic events [57]. Anyway, it should be specially pinpointed that the rate of hospitalization for heart failure was increased [58, 81–83]—in contrast to the findings reported for sitagliptin [60, 79]. Moreover, the risk of heart failure hospitalization was augmented irrespective of age category [84], being highest among patients with elevated levels of natriuretic peptides, previous heart failure, or chronic kidney disease [85].

#### Linagliptin

Linagliptin is a potent gliptin with a xanthine-based molecular structure [86–88]. It inhibits DPP4 competitively and reversibly, showing a slow rate of dissociation from the active center of the DPP4 enzyme molecule, and regarding selectivity towards DPP4 in comparison to other enzymes of the DPP family, it is 40,000-fold higher towards DPP-4 than towards DPP-8 and >10,000-fold higher towards DPP-9 [89]. Linagliptin is rapidly absorbed after oral administration with a T_max_ of 0.7–3 h that does not differ between healthy and T2DM subjects after single or multiple doses [90–92]. A pre-specified patient-level pooled analysis of all available double-blind, randomized, controlled trials evaluating the cardiovascular safety of the drug has been recently published [54]. The study encompassed 19 trials of ≥12 weeks’ duration, including nearly 9500 subjects receiving linagliptin versus placebo or another active treatment. Out of these patients, about 5850 received linagliptin; most of them 5 mg daily. The comparators were glimepiride, voglibose and placebo. The primary end point was time to the first occurrence of any components of the 4P-MACE composite, i.e. cardiovascular death (including fatal stroke and fatal MI), non-fatal MI (excluding silent MI), non-fatal stroke or hospitalization for unstable angina pectoris. The main limitation of this study is the mean duration of the included trials, considerably shorter than the recommended by the FDA [24], reducing so the extent of interpretations that can be made. Anyway, the incidence rate of 4P-MACE was 13.4 events per 1000 patient-years for linagliptin-treated patients compared with 18.9 in the active comparator group; therefore it should be pointed out that linagliptin was not associated with an increase in cardiovascular risk.

Two ongoing trials will provide a more definitive answer on the cardiovascular safety profile of linagliptin. The first is the CAROLINA trial [93, 94], started in 2010, that has randomized more than 6000 patients with early T2DM and predominantly medium cardiovascular risk, to treatment with either linagliptin or glimepiride. Therefore, it is the first head-to-head outcome trial of a gliptin versus an active comparator that is sufficiently powered to unveil potential differences in MACE occurrence between treatment groups [54]. The second one is the CARMELINA trial, initiated in 2013 and aimed to investigate the long term impact on cardiovascular morbidity, mortality and renal function in a selected population of 8300 patients with T2DM and renal compromise, comparing outcomes against placebo, on a background of standard of care [95]. The results of both trials are expected in 2018. For the time being, the available information regarding linagliptin and kidney function is encouraging [96–98]. In addition, it has been documented that linagliptin ameliorates cardiovascular injury in salt-sensitive hypertensive rats independently of blood glucose and blood pressure [44], and also attenuates neointima formation after vascular injury and vascular smooth muscle cells (VSMC) proliferation beyond its glucose-lowering effect [56].

#### Alogliptin

Alogliptin is a pyrimidinedione derivative highly selective gliptin, rapidly absorbed, with a mean time to maximum concentration (C_max_) of approximately 1–2 h [99–101]. Preliminary clinical studies reported favorable results [102, 103]. The EXAMINE trial was programmed in order to assess its cardiovascular safety in T2DM patients with acute coronary syndrome [104]. It was a multicentre, double-blind trial, into which 5380 subjects—enrolled from 49 countries—who underwent such an event event in the previous 15–90 days were randomly assigned to alogliptin 25 mg daily or placebo plus standard treatment for diabetes and cardiovascular disease prevention. The results have been recently published [105]. The pre-specified MACE endpoint was all-cause mortality, non-fatal MI, non-fatal stroke, urgent revascularization for unstable angina syndrome, and hospitalization for heart failure. Alogliptin neither increased cardiovascular morbidity or mortality, nor worsened pre-existing heart failure, including in those patients with a very recent acute coronary syndrome, after a median duration treatment of 18 months [106, 107]. In addition, assessment of N-terminal pro-BNP (NT-pro-BNP) concentration from baseline to 6 months did not reveal any significant changes.

The safety of the co-administration of alogliptin and pioglitazone has been documented in both clinical [48, 107] and experimental [108] settings. Moreover, additional beneficial effects have been described. The drug attenuates arterial inflammation and neointimal formation after injury in low-density lipoprotein receptor-deficient mice [109] and—besides the expected decrease in glucose and hemoglobin A1c—leads also to a decrease in serum lipids in humans [110]. A preliminary report indicates that in the postprandial state lipemia diminishes, and endothelial dysfunction improves in non-diabetic subjects [111]. An anti-arrhythmogenic effect was described in rabbits via augmentation of atrial angiogenesis [112].

#### Vildagliptin

Vildagliptin is a cyanopyrrolidine derivative [113–115]. A large retrospective meta-analysis of prospectively adjudicated cardiovascular events has been recently published, including data from 40 double-blind, randomised-controlled phase III and IV studies encompassing about 17,500 patients (mean age 57 years) receiving vildagliptin 50 mg once and twice daily, or comparators [116]. The primary endpoint was occurrence of any MACE. The duration of the trials was very variable, lasting from 12 up to 104 weeks, and the average length of treatment was about 1 year. The results indicated that the medication was not associated with an increased risk for adjudicated MACE relative to comparators. Moreover, the event rates for new onset of heart failure or hospitalization for its aggravation were relatively low (0.4 %) and similar both groups.

Another vildagliptin trial is the VISUAL study [117]. The study was aimed to compare the efficacy and safety of adding vildagliptin with sulfonylurea dose-increasing as an active comparator in patients with inadequately controlled T2DM using metformin plus sulfonylurea in a real clinical setting. The study demonstrated that the vildagliptin add-on group exhibited no clinically relevant weight gain and had a lower incidence of hypoglycemia compared with the sulfonylurea group, and the addition of vildagliptin to sulfonylurea could be considered as a treatment option prior to intensification with insulin [118].

In rodent models, vildagliptin enhanced blood flow recovery and capillary density in ischemic limbs with accompanying increases in endothelial nitric-oxide synthase [119] and prevented left ventricular hypertrophy caused by continuous beta-adrenergic stimulation by isoproterenol [120].

#### Anagliptin

Like saxagliptin and vildagliptin, anagliptin is a cyanopyrrolidine derivative possessing a prolylprolinenitrile scaffold [113, 121], available for clinical use in Japan since 2012. Since this compound is not generally used in countries other than Japan, there is a paucity of reports investigating its effects. The recommended dose of is 200 mg daily (100 mg bid), albeit increases in the dose up to 400 mg daily have been approved in cases in which the blood glucose-lowering effect is unsatisfactory [52].

Besides its hypoglycemic effects, a pooled analysis of data obtained from Phase III trials, the serum LDL-cholesterol, triglycerides, total cholesterol and non-high-density lipoprotein-cholesterol levels were significantly reduced after the administration of anagliptin at doses of 200 or 400 mg [122]. Moreover, it has been demonstrated in a model of apoE-deficient mice that anagliptin reduced the area of atherosclerotic lesions, suggesting thus a potential anti-atherogenic action via direct inhibition of smooth muscle cell proliferation and inflammatory reaction of monocytes [123].

#### Teneligliptin

Teneligliptin is a prolylthiazolidine [61] approved for the treatment of T2DM in Japan in 2012 and in South Korea in 2014 [124, 125]. A 4-week, randomized, double-blind, placebo-controlled trial showed a significantly improved 24-h blood glucose control [126], and a subsequent pooled post hoc analysis including more than 700 patients provides evidence of the safety and efficacy of a 52 weeks mean follow-up of use of teneligliptin 20 mg daily as monotherapy or in combination with sulfonylurea, glinide, biguanide or α-glucosidase inhibitor in Japanese patients with T2DM [127]. The dose can be increased up to 40 mg per day [128]. Teneligliptin was also used as an initial monotherapy for drug-naive newly diagnosed T2DM subjects. It activated beta-cell function and decreased insulin resistance, being rather effective in reducing both fasting blood glucose levels and HbA1c. However, a significant increase in uric acid concentration was observed in some patients [129].

Regarding the cardiovascular effects, it should be pinpointed that QT/QTc evaluations were performed for this compound [128]. No QT prolongations were detected with 40 mg daily of teneligliptin, which is the maximal dose in usual clinical practice. Anyway, a mild QTc transient prolongation was documented while using supraclinical dosages. Therefore, caution is needed if the drug is used for a long period or in co-administration with medications known to cause QT prolongation on their own [128]. On the other hand, teneligliptin treatment was associated with improvements in left ventricular function—particularly diastolic—and endothelial functions, as well as with an increase in serum adiponectin levels [130].

### Mechanistic construal

**Fig. 1:**
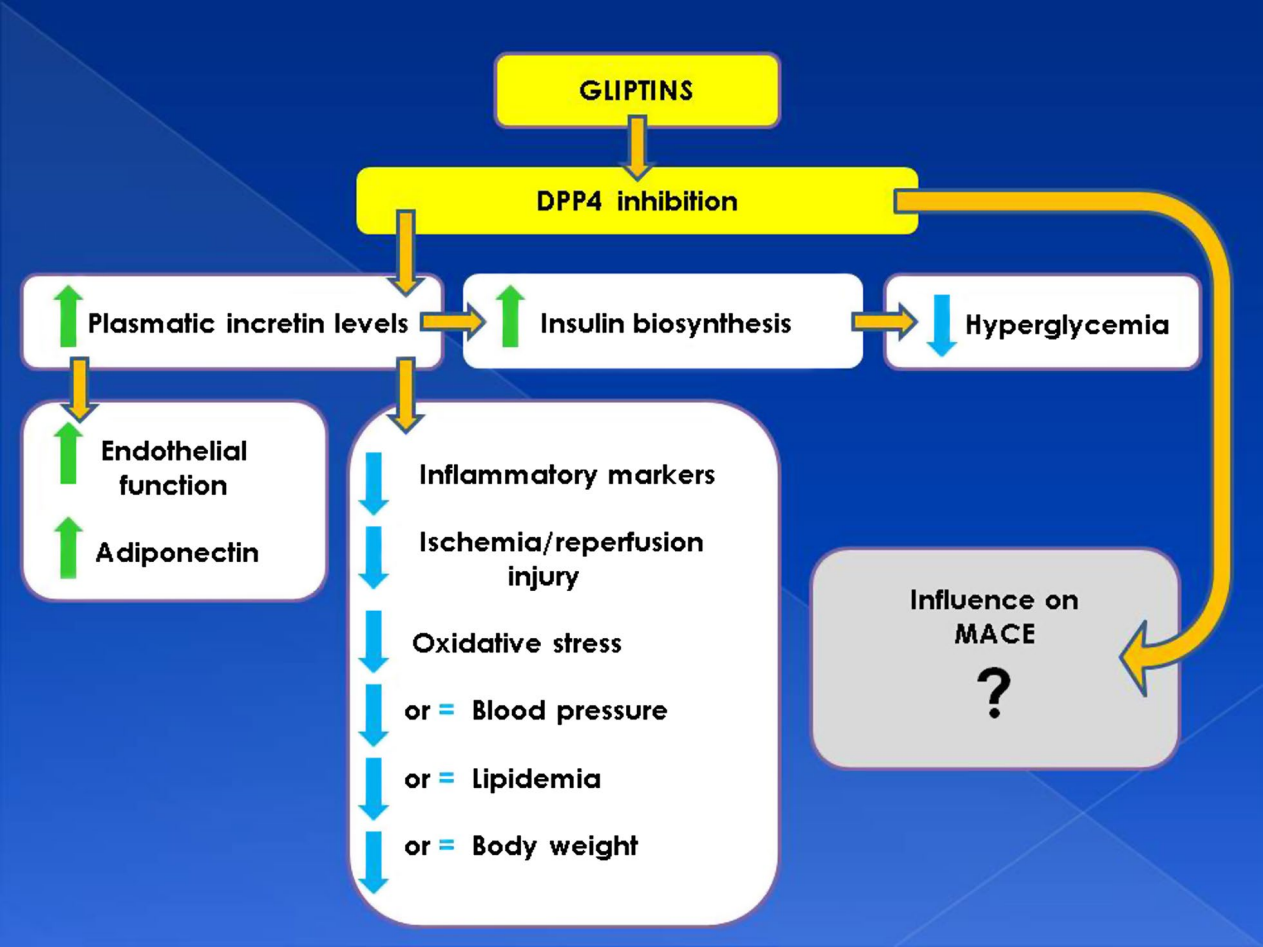
Schematic depiction of the main biochemical and clinical effects of DPP4 (dipeptidyl peptidase-4) inhibition. While both the increasing (*green arrows*) and decreasing (*blue arrows*) effects seem to be favorable, the overall influence on MACE (major adverse cardiovascular events) remains uncertain

Which could be the molecular mechanisms for the favorable results of DPP4 inhibition in experimental settings? Several responses can be outlined. DPP4 is a transmembrane glycoprotein that cleaves N-terminal dipeptides from many substrates. It is ubiquitously expressed, including several growth factors, hormones, neuropeptides, and chemokines [30, 33, 131]. Using a proteomics approach, human adipocytes’ secretome has been unveiled, identifying DPP4 as a newfangled adipokine, produced particularly by fully differentiated adipocytes [132, 133]. Anyway, it should be emphasized that the main divergence with many other adipokines is that DPP4 is not directly secreted by adipocytes but released from the plasma membrane as soluble DPP4 following proteolytic cleavage [32, 134]. Soluble DPP4 is a striking activator of both mitogen-activated protein kinases (MAPK) and nuclear factor kappa B (NF-κB); these pro-inflammatory signaling pathways lead to proliferation and atherogenic transformation of VSMC [133]. A DPP-4 inhibitor exerts anti-inflammatory effects on macrophages and adipocytes, being able to suppress NF-κB activation [135]. In this context, soluble DPP4 also induces activation of extracellular-signal-regulated kinases (ERKs, a specific subset of the MAPK family) in VSMC, which was partially blocked by DPP4 inhibition [123]. The main effects of this inhibition are depicted in Fig. 1.

DPP4 consistently impaired insulin signaling at the level of protein kinase B (the so-called Akt) in several primary cell types like adipocytes, skeletal muscle, and VSMC [132]. In addition, it has been shown that while a trend for higher DPP4 expression in visceral fat is present lean subjects, in obese subjects DPP4 is significantly higher in visceral adipose tissues than in its subcutaneous counterparts [132]. DPP4 expression in both adipose depots is significantly higher in obese compared with lean subjects, and its levels positively correlate with body mass index (BMI), subcutaneous and visceral adipocytes size, insulin, and leptin [132, 136], whereas a negative correlation with age and adiponectin was found [132, 137]. High DPP4 activity might induce subsequent accumulation of body fat and increased BMI [138], and in turn long-term obesity and lipid accumulation will lead to insulin resistance and hyperglycemia via abnormally obesity-linked down-regulation of adiponectin [139]. Moreover, an increased DPP4 production from adipocytes is observed in both type 1 and T2DM [140]. Together, all these biochemical facts represent a mechanistic construal reflecting a solid scaffold for the alleged beneficial cardiovascular outcomes obtained by inhibition of DPP4 activity.

Therefore, besides their glucose-lowering activity, gliptins have an intrinsic manifold cardiovascular impact. This impact is rooted in the fact that the presence of GLP-1 receptors in human cardiac myocytes has been demonstrated two decades ago [141] and subsequently confirmed in later years [142, 143], boosting the interest of researchers to properly establish the relevance of the gliptin-induced changes in clinical settings. As mentioned above, the changes are explained by reasonable molecular mechanisms and seem to be mainly beneficial. They include improvement of endothelial function, decrease of inflammatory markers, reduction of ischemia/reperfusion injury in experimental models, prevention of left ventricular remodeling, modest decreases in blood pressure and lipidemia, and a trend to lower MACE incidence.

### Controversial issues

Unfortunately, the above mentioned favorable features are counterbalanced by other ones. For instance, in addition to inactivate the incretin hormones, DPP4 can also affect the orexigenic hormone neuropeptide Y, and its inhibition by gliptines enhances antilipolytic action in human adipose tissue leading to further accumulation of body fat [144, 145]. Additional important points represent a source of concern, such as the significantly higher rate of hospitalization for heart failure with saxagliptin reported in the SAVOR-TIMI 53 trial [58], the fact that the research regarding dutogliptin—initially considered a promising selective DPP4 inhibitor [146] was abruptly discontinued without giving any reason [63] and the finding of hyperuricemia in patients treated with teneligliptin [129]. A recent assessment of the results of the EXAMINE trial [105] points out that the rate of heart failure was increased in patients on alogliptin who had no previous history of this disorder [147]. Moreover, the co-administration of gliptins with ACE-inhibitors or angiotensin receptor blockers maybe associated with severe angioedema [148, 149], despite favorable metabolic effects in an experimental model [74].

Several explanations can be hypothesized regarding these conflicting results. In first place, gliptins are multi-target compounds, and therefore their activity is connected with the inhibition of various substrates, leading to undesirable effects [150]. Moreover, DPP4 inhibitors may exert differential effects on substrate activity in a diabetic versus a normoglycemic setting [151] and chronic treatment with gliptins exerts progressive changes in metabolic parameters beyond those detected in single-dose administration studies [152]. Finally, most of the trials were not long enough to comply with the FDA recommendations regarding cardiovascular outcomes [24].

Regardless the differences in their chemical structure and metabolic pathways, gliptins as a group constitute a widely accepted therapy for the management of T2DM, usually as a second-line medication [27, 42, 153–156]. It has neutral effects on weight [157, 158]; oral administration and low incidence of hypoglycemia are important advantages. On the other hand, while experimental and preliminary clinical studies on several surrogate end-points suggest that gliptins can exert cardioprotective actions, the results of placebo-controlled phase IV trials have been rather disappointing so far. In clinical settings, several trials—notably the longer one, with sitagliptin—did not show an increased risk for ischemic events [60], but the encouraging results from basic science were not yet translated into clinical evidence, probably due the pleiotropic enzymatic effects of DPP4 [159, 160]. The relatively rapid FDA approval of sitagliptin for clinical use has been criticized [161], post-marketing reports reveal safety aspects that need further investigation [162], and it has even been suggested to restrict the use of gliptins to Phase IV clinical trials until cardiovascular safety is clearly established [163].

## Conclusion

For the time being, a definite relationship between gliptins treatment and improved cardiovascular outcomes remains uncertain and needs yet to be proven.

## Abbreviations

BMI: body mass index
DPP4: dipeptidyl peptidase-4
FDA: Food and Drug Administration
GIP: glucose-dependent insulinotropic polypeptide
GLP-1: glucagon-like peptide-1
MACE: major adverse cardiovascular events
MI: myocardial infarction
NF-κB: nuclear factor kappa B
T2DM: type 2 diabetes mellitus
VSMC: vascular smooth muscle cells
